# Key features of invasive pneumococcal isolates recovered in Lima, Peru determined through whole genome sequencing

**DOI:** 10.1016/j.ijmm.2017.07.008

**Published:** 2017-10

**Authors:** Paulina Hawkins, Erik Mercado, Sopio Chochua, Maria E. Castillo, Isabel Reyes, Eduardo Chaparro, Rebecca Gladstone, Stephen D. Bentley, Robert F. Breiman, Benjamin J. Metcalf, Bernard Beall, Theresa J. Ochoa, Lesley McGee

**Affiliations:** a Emory University, Atlanta, USA; b Centers for Disease Control and Prevention, Atlanta, USA; c Universidad Peruana Cayetano Heredia, Lima, Peru; d Instituto Nacional de Salud del Niño, Lima, Peru; e Hospital de Emergencias Pediátricas, Lima, Peru; f Hospital Cayetano Heredia, Lima, Peru; g Wellcome Trust Sanger Institute, Cambridge, UK

**Keywords:** *S. pneumoniae*, Whole genome sequencing, Antimicrobial resistance

## Abstract

Before PCV7 introduction, invasive pneumococcal disease (IPD) was responsible for approximately 12,000–18,000 deaths annually among children <5 years in Latin America. In Peru, PCV7 was introduced in 2009. We used whole genome sequencing to deduce key features of invasive strains collected in Lima, Peru from 2006 to 2011. We sequenced 212 IPD isolates from 16 hospitals in Lima pre (2006–2009; n = 133) and post (2010–2011; n = 79) PCV7 introduction; 130 (61.3%) isolates were from children ≤ 5 years old. CDC’s *Streptococcus* lab bioinformatics pipeline revealed serotypes, sequence types (STs), pilus genes, PBP types and other resistance determinants. During the pre-PCV7 period, serotype 14 was the most common serotype (24.8%), followed by 6 B (20.3%), 19F (10.5%), and 23F (6.8%). Post-PCV7, the proportion of PCV7 serotype 6 B decreased significantly (to 6.3%), while 19F (16.3%), 14 (15.0%), 23F (7.5%), and 19A (7.5%) were the most common serotypes; only serotypes 3 and 10A increased significantly. Overall, 82% (n = 173) of all isolates carried at least one resistance determinant, including 72 (34%) isolates that carried resistance determinants against 3 or more antimicrobial classes; of these 72 isolates, 56 (78%) belonged to a PCV7 serotype. Eighty-two STs were identified, with 53 of them organized in 14 clonal complexes. ST frequencies were distributed differently pre and post-PCV7 introduction, with only 18 of the 57 STs identified in years 2006–2009 isolates also observed in years 2010–2011 isolates. The apparent expansion of a 19F/ST1421 lineage with predicted *β*-lactam resistance (PBP type 13:16:20) and carrying resistance determinants against four additional antimicrobial classes was observed.

## 1 Introduction

Infections caused by *Streptococcus pneumoniae* include serious conditions such as meningitis, bacteremia, and pneumonia as well as less severe conditions such as sinusitis and otitis media. The World Health Organization (WHO) estimated that pneumococcal infections caused 476,000 (5%) deaths among HIV-negative children under five years of age during 2008 (WHO, 2008). *S. pneumoniae* includes >90 serotypes. Prior to introduction of pneumococcal conjugate vaccines, only 11 of these serotypes accounted for the majority of invasive pneumococcal disease (IPD) in children worldwide (Johnson et al., 2010).

The first pneumococcal conjugate vaccine covered 7 serotypes (PCV7: 14, 6B, 19F, 23F, 4, 9 V, 18C) and was licensed in 2000, followed by PCV10 (PCV7 serotypes plus 1, 5, and 7F) in 2009, and PCV13 (PCV10 serotypes plus 3, 6A, and 19A) in 2010. These vaccines have been shown to be highly effective for protecting infants and young children against IPD caused by vaccine serotypes and to diminish acquisition of carriage by serotypes included in the vaccine (WHO, 2012).

In Latin America and the Caribbean, pneumococcal infections were estimated to account for 12,000–18,000 deaths, 327,000 cases of pneumonia, 4000 cases of meningitis and 1229 cases of sepsis each year in children aged under five years before vaccine introduction (Constenla et al., 2007). In Peru, PCV7 was introduced into the national immunization program in 2009 as a 3 dose schedule at 3, 5, and 12 months of age; it was replaced by PCV10 in late 2011, given in three doses at 2, 4, and 12 months of age. A catch-up campaign included two doses for unvaccinated children between 12 and 24 months of age and a single dose for children 2–5 years old with a comorbidity. In 2007, it was estimated that PCV7 would cover 62% of the circulating isolates recovered from children under 6 years of age in Peru, while PCV10 would cover 71% (Ochoa et al., 2007).

The aim of this study was to determine serotypes, genotypes, and resistance determinants of IPD isolates relevant to current conjugate vaccine evaluation and prospective prevention efforts in Lima, Peru. A secondary aim was to evaluate the performance of the automated whole genome sequence (WGS) bioinformatics pipeline developed by the *Streptococcus* lab at CDC in deducing these key features among these isolates, since it had only been previously employed with U.S. IPD isolates (Metcalf et al., 2016a, Metcalf et al., 2016b, Li et al., 2016).

## 2 Materials and methods

We extracted DNA from 212 IPD isolates from children and adults in 16 hospitals in Lima, pre (2006–2009; n = 133) and post (2010–2011; n = 79) PCV7 introduction, as part of a passive surveillance study conducted by the Peruvian Group on Pneumococcal Research (Grupo Peruano de Investigación en Neumococo, GPIN). All isolates were serotyped by latex agglutination and the Quellung reaction employing CDC antisera. E-tests (Biomérieux, Marcy l’Etoile, France) were performed on select isolates.

Whole genome sequencing was performed at the Sanger Institute using the Illumina HiSeq 2500 system, as part of the Global Pneumococcal Sequencing project (www.pneumogen.net), and data submitted to the European Nucleotide Archive (accession numbers in Table S1). Sequences were analyzed using the CDC’s *Streptococcus* lab pneumococcal typing pipeline to identify serotypes, sequence types (STs), pilus genes, transpeptidase domain amino acid sequences from penicillin-binding proteins (PBPs) 1a, 2b, and 2x, and other resistance features (https://github.com/BenJamesMetcalf/Spn_Scripts_Reference) (Metcalf et al., 2016a, Metcalf et al., 2016b). Non-susceptibility to 6 different *β*-lactams was predicted by assigning a PBP type as previously described (Metcalf et al., 2016a, Metcalf et al., 2016b, Li et al., 2016), and correlating this PBP type with phenotypically measured MIC values for isolates with the same type (http://www.cdc.gov/streplab/mic-tables.html), based on current CLSI guidelines (CLSI, 2015). Penicillin susceptibility, intermediate resistance, and resistance were defined as MIC of ≤0.06, 0.12–1.0, and ≥2.0 mg/L, respectively. For cefotaxime and ceftriaxone, susceptibility, intermediate resistance, and resistance were defined as MIC of ≤1.0, 2.0, and ≥4.0 mg/L, respectively. Cefuroxime susceptibility, intermediate resistance, and resistance were defined as MIC of ≤0.5, 1.0, and ≥2.0 mg/L, respectively. Amoxicillin susceptibility, intermediate resistance, and resistance were defined as MIC of ≤2.0, 4.0, and ≥8.0 mg/L, respectively. Meropenem susceptibility, intermediate resistance, and resistance were defined as MIC of ≤0.25, 0.5, and ≥1.0, respectively. For previously unreported PBP types, MIC values against penicillin and cefotaxime were determined using E-tests (Biomérieux, Marcy l’Etoile, France). Contingency tables and a chi-squared test (or a Fisher’s exact test) were used to determine significance of associations (at α = 0.05).

## 3 Results and discussion

### 3.1 Serotype and sequence type distribution

The majority of the samples were isolated from blood (57%), CSF (24%), or pleural fluid (10%); 130 (61.3%) were isolated from children age 5 and under (101 pre-PCV7 and 29 post-PCV7). The most common clinical manifestations were pneumonia (45.3%) and meningitis (29.2%); the proportions did not differ significantly pre and post-PCV7 introduction. There was a significant association between serotype 14 and pneumonia (p = 0.01), as well as between serotype 5 and pneumonia, where 86% of isolates (6/7) were obtained from patients with pneumonia (five of those patients were age 2 or younger). Thirty-four serotypes were identified among the 212 isolates, with serotype 14 being the most common (24.8%), followed by 6 B (20.3%), 19F (10.5%), and 23F (6.8%) pre-PCV7 introduction; 19F (16.3%), 14 (15.0%), 23F (7.5%), and 19A (7.5%) were the most common serotypes post-PCV7. We observed almost perfect concordance between conventional and WGS-based serotype determination. The only exception was an isolate that was non-typable by quellung, but was determined to be serotype 13 by WGS-based serotyping.

**Fig. 1:**
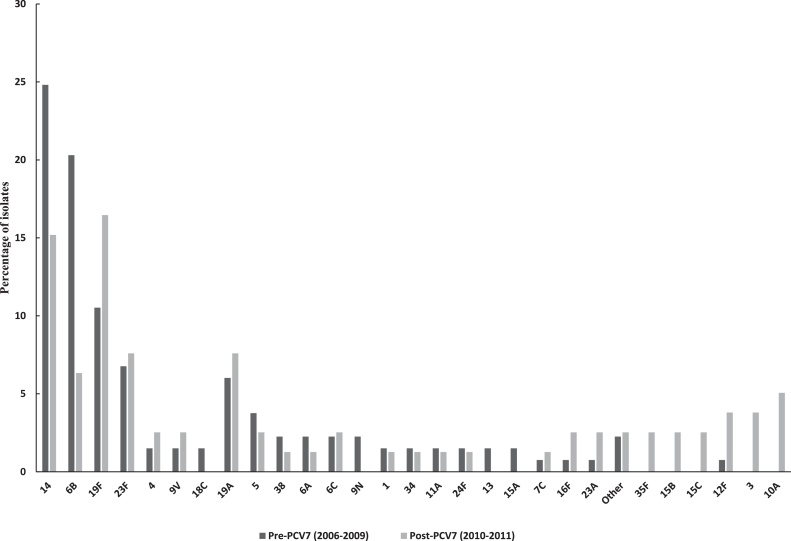
Serotype distribution of all isolates in the study, before and after PCV7 introduction. Dark grey bars represent pre-PCV7 isolates. Light grey bars represent post-PCV7 isolates. Other: 18F, 23B, 7F, 39 and 19B. Stars represent significant changes in serotype proportions after PCV7 introduction.

The proportion of PCV7 serotype 6 B decreased significantly (to 6.3%, p = 0.003) after vaccine introduction, as did that of serotype 14 (to 15.2%, p = 0.05). The proportion of serotypes 23F, 4, and 9 V changed very little. On the other hand, the proportion of 19F increased slightly by 5.9% (p = 0.11), possibly due to serotype-specific PCV7 effectiveness (lowest for 19F at 87%) and the short time period between PCV7 introduction and our observations; it has been shown that serotype 19F isolates can still persist several years after vaccine introduction (Metcalf et al., 2016a). The proportion of non-PCV7 serotypes 3 and 10A increased significantly (p = 0.05 and p = 0.02, respectively) (Fig. 1).

**Fig. 2:**
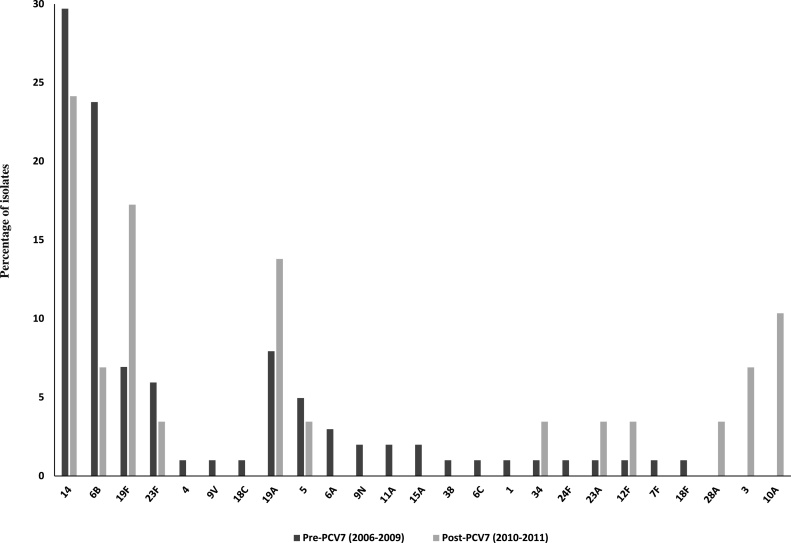
Serotype distribution of isolates obtained from children age 5 and under, before and after PCV7 introduction. Dark grey bars represent pre-PCV7 isolates. Light grey bars represent post-PCV7 isolates. Stars represent significant changes in serotype proportions after PCV7 introduction.

Among children age 5 and under, 14 (29.7%), 6 B (23.8%), and 19F (6.9%) were the most common serotypes recovered pre-PCV7; this distribution is consistent with previous reports (Constenla et al., 2007), including a study on a subset of these isolates (Ochoa et al., 2010) and the PAHO’s SIREVA II program findings in Peru for 2008 (OPS, 2009). The overall proportion of PCV7 serotypes (69.3%) was in concordance with previous estimates (Ochoa et al., 2007). After PCV7 was introduced, serotype 14 decreased to 24.1% (p = 0.28) and 6 B decreased to 6.9% (p = 0.02), while the proportions of 19F and 19A isolates increased, but not significantly (p = 0.09 and p = 0.17, respectively), and the proportion of non-PCV7 serotypes 3 and 10A increased significantly (p = 0.05 and p = 0.01, respectively) (Fig. 2). In contrast, the SIREVA II 2012 report showed that 19A was the most common serotype among isolates recovered from children under 5 years old in Peru (OPS, 2013a). Replacement by non-vaccine serotypes, such as 19A, was also observed in the United States (Hicks et al., 2007, Moore et al., 2008) and other countries (WHO, 2010) after PCV7 introduction.

Eighty-two STs were identified among all isolates, with 53 organized in fourteen clonal complexes (CC) (Table 1). The most prevalent CCs were CC156 pre-PCV7 and CC1421 post-PCV7 introduction. Of the 57 STs identified among 2006–2009 isolates, only eighteen remained after PCV7 introduction, along with 26 unique STs from 2010 to 2011 isolates. Pre-PCV7, CC156 was most commonly associated with serotype 14 and thus expectedly declined along with this serotype after PCV7 introduction; this was also the case for ST15 and ST25. ST1121, ST90 and ST135 declined along with serotype 6 B after PCV7 introduction, while CC1421 increased alongside serotype 19F. No significant capsular switching or clonal shift was observed among these isolates, probably due to the limited post-PCV7 period covered by this study.

**Table 1:** Clonal complexes identified, with associated serotypes.

Clonal complex	ST	n	Pre	Post	Serotypes (n)
CC156	156	38	28	10	14 (36), 9V (1), 23F (1)
(PMEN3)	7432	1		1	14 (1)
	5458	1	1		14 (1)
CC1121	1902	1		1	34 (1)
	1121	3	3		6B (3)
	5619	1	1		6B (1)
	5628	1	1		6B (1)
	5472	3		3	10A (3)
CC1421	1421	14	6	8	19F (14)
(PMEN14)	11396	1		1	19F (1)
	5459	1	1		19F (1)
	320	4	2	2	19A (4)
	7132	1	1		19F (1)
	5676	1		1	19F (1)
CC81	81	8	4	4	23F (5), 19F (3)
(PMEN1)	1591	1	1		19F (1)
	9904	1		1	19F (1)
	66	4	3	1	9N (2), 19A (2)
	5593	2	2		13 (2)
CC90	90	6	5	1	6B (6)
(PMEN2)	1624	1	1		6B (1)
	273	1	1		6A (1)
CC15	15	3	3		14 (3)
(PMEN9)	25	2	2		14 (2)
	6144	1	1		14 (1)
	5460	1		1	19A (1)
	9054	1		1	14 (1)
	6048	1	1		19A (1)
	6809	1	1		7C (1)
	5468	1		1	7C (1)
CC218	218	4	1	3	12F (4)
(PMEN34)	5455	1	1		7F (1)
	6143	1	1		18C (1)
CC135	135	3	3		6B (3)
	1662	1	1		6B (1)
	1876	1		1	6A (1)
CC276	276	3	3		19A (3)
(PMEN32)	6741	1		1	24F (1)
CC5448	5448	1	1		15A (1)
	5452	1	1		19A (1)
	5453	1	1		15A (1)
CC505	9060	1		1	3(1)
	505	2		2	3 (2)
	5616	1	1		9N (1)
CC1131	1131	2		2	19A (2)
(PMEN26)	6140	1	1		23B (1)
	338	1		1	23A (1)
CC5625	5625	7	6	1	6B (7)
	5626	1	1		6B (1)
	5623	1	1		6A (1)
	5449	4	3	1	6B (4)
CC6139/5581	6139	1	1		24F (1)
	5581	1	1		24F (1)
Singletons (PMEN15)	242	9	6	3	23F (9)
(PMEN19)	289	7	5	2	5 (7)
	1292	5	3	2	6C (5)
	5475	4	3	1	38 (4)
(PMEN20)	315	3	1	2	6B (3)
	646	3	3		19F (3)
(PMEN29)	615	3	2	1	1 (3)
	439	2	1	1	23A (2)
	5465	2		2	4 (2)
	6149	2	1	1	16F (2)
	3669	4		4	15B (2), 15C (2)
	206	2	2		4 (2)
	280	3	1	2	9V (3)
	Other	16	8	8	
**Total**		**212**	**133**	**79**	

There were several PMEN clones (www.pneumogen.net/pmen/) represented among these isolates: PMEN1, PMEN2, PMEN3, PMEN14, PMEN15, PMEN19, PMEN20, and PMEN26. Most notably, PMEN3 (Spain^9V^-ST156) was represented by 38 ST156 isolates (36 serotype 14, one 9 V, and one 23F); while PMEN14 (Taiwan^19F^-ST236) was represented by 14 19F/ST1421 (double locus variant) isolates, in addition to the previously reported ‘vaccine escape’ 19A/ST320 isolates (Metcalf et al., 2016a, Moore et al., 2008, Beall et al., 2011).

### 3.2 Antimicrobial resistance

The capability of a WGS-based approach to accurately and reliably predict antimicrobial phenotypes has been previously shown to be an acceptable substitute for broth dilution testing (Metcalf et al., 2016b, Li et al., 2016). By a WGS-based assessment of resistance, 157 (74.1%) isolates were predicted to be non-susceptible to cotrimoxazole, 105 (49.5%) non-susceptible to *β*-lactams, 84 (39.6%) resistant to tetracycline, 76 (35.8%) resistant to erythromycin, 53 (25%) resistant to clindamycin, and 21 (9.9%) resistant to chloramphenicol, by identifying genetic determinants. The proportion of isolates predicted as non-susceptible/resistant to each antimicrobial class did not change significantly from the pre-PCV7 to the post-PCV7 period. These predicted rates were consistent with reported rates for neighboring countries (Constenla et al., 2007; OPS, 2013b) pre-PCV introduction: in Ecuador, the rates of erythromycin and cotrimoxazole resistance were similar (23% and 67%, respectively), while the rate of penicillin non-susceptibility was lower (13%); in Brazil, the rates of penicillin and cotrimoxazole non-susceptibility were similar (37% and 73%, respectively), while the rate of erythromycin resistance was lower (11%).

Of the 157 isolates that were predicted as non-susceptible to cotrimoxazole, all contained 1–2 codon insertions within the *fol*P gene (intermediate phenotype, MIC 1–2 μg/ml), while 125 of them (79.6%) also contained changes in the *fol*A (I100L) gene (resistant phenotype, MIC ≥4 μg/ml). Of the 76 isolates predicted as resistant to erythromycin, 23 (30.3%) were positive for *erm*B alone, 23 (30.3%) for *mef*A alone, and 30 (39.4%) for *erm*B plus *mef*A. In addition, 84 isolates were positive for *tet*M and 21 isolates for the *cat* gene. One isolate contained changes in the *gyr*A and *par*C genes and was resistant to levofloxacin and ciprofloxacin by broth microdilution (MIC >8 μg/ml and MIC > 4 μg/ml, respectively); this isolate (GPS_P2272, 19F/ST1421) was obtained from a blood sample from a 5 year old patient with pneumonia and was also resistant to cotrimoxazole, erythromycin, clindamycin, chloramphenicol, tetracycline, and several *β*-lactams. Of the 105 isolates that were predicted as non-susceptible to *β*-lactams: all were predicted as non-susceptible to penicillin, 46 non-susceptible to ceftriaxone, 78 non-susceptible to cefuroxime, 23 non-susceptible to cefotaxime, and 5 non-susceptible to meropenem; 57 isolates were predicted as non-susceptible to at least three of the *β*-lactams tested, including four isolates non-susceptible to all six (all 19A/ST320). Predicted penicillin MICs ranged from 0.75 to 4 μg/ml among these 57 isolates.

Overall, 81.6% of the 212 isolates were positive for at least one resistance determinant (82% pre vs 81% post-PCV7 introduction), including 72 (34%) isolates that were positive for resistance determinants against three or more antimicrobial classes; of these 72, 77.7% belonged to one of the PCV7 serotypes and 11.1% were serotype 19A. Of the 133 pre-PCV7 isolates, 32.3% (n = 43) were predicted as resistant to at least 3 antimicrobial classes, of these: 27.9% (n = 12) were serotype 19F, 27.9% (n = 12) were 6B, 11.6% (n = 5) were 14, and 9.3% (n = 4) were 23F. After PCV7 introduction, the proportion of multidrug resistance (MDR) did not change significantly (p = 0.35), with 36.7% (n = 29) of the isolates predicted as resistant to at least 3 antimicrobial classes: 37.9% (n = 11) of these isolates belonged to serotype 19F, 10.3% (n = 3) to serotype 14 (ST156), 10.3% (n = 3) to 6B, and 10.3% (n = 3) to serotype 23F.

Among children age 5 and under, the proportion of isolates resistant to at least 3 antimicrobials decreased from 34.7% before (n = 35) to 24.1% after (n = 7) PCV7 introduction (p = 0.14), mostly due to a drop in serotype 6 B isolates. The persistence of MDR observed among our isolates after PCV7 introduction could be explained by the persistence of resistant 19F (mostly CC1421) isolates, as there was a strong association between MDR and serotype 19F, both before (OR = 17.36, p < 0.001) and after (OR = 5.84, p = 0.003) PCV7 introduction. Thus, it is likely that prevalence of MDR decreased in later years, as PCV coverage reportedly increased from 37.9% in 2009–95% in 2012 (Suarez et al., 2016).

### 3.3 PBP types

Thirty-nine new PBP allele combinations (types) were identified among 42 (19.8%) isolates: 29 (74.4%) in isolates from the pre-PCV7 period and 10 (25.6%) in isolates from the post-PCV7 period; 19 (48.7%) of the 39 combinations were associated with non-susceptibility to penicillin. Identifying many new allele combinations was expected, as the PBP database used for analyses only contained isolates from the United States. As more PBP genes are sequenced from other regions of the world, new PBP types will continue to be identified. PBP types identified among our isolates associated with non-susceptibility to *β*-lactams correlated strongly with specific serotype and STs, which is expected from the small sampling in this study (Li et al., 2016). Of the eight most frequent combinations, seven were associated with a unique clonal complex (Table 2).

**Table 2:** Association between 8 most common ST/CC and PBP type among isolates with β-lactam non-susceptibility.

PBP type (1A:2B:2X)	n	ST/CC	p-value	Serotypes	PEN MIC (μg/ml)	TAX MIC (μg/ml)	CFT MIC (μg/ml)	AMO MIC (μg/ml)	CFX MIC (μg/ml)	MER MIC (μg/ml)
15:12:18	23	CC156	<0.0001	14(21), 9V(1), 23F(1)	4	1	2	2	>2	0.5
	10	CC81	0.002	19F(5), 23F(5)						
13:16:20	14	CC1421	<0.0001	19F	2	1		2	>2	0.5
45:12:63	12	ST156	<0.0001	14	4	2		2	>2	0.5
13:11:16	4	ST320	<0.0001	19A	4	2	2	8	>2	1
8:67:103	4	CC135	<0.0001	6B	0.5	0.25	≤0.25	0.25	≤0.5	≤0.06
34:57:56	3	ST90	<0.0001	6B	1	0.5		0.25	>2	0.12
2:53:77	3	ST315	<0.0001	6B	0.12	≤0.06	0.12	0.25	≤0.5	≤0.06
96:113:185^a^	3	ST646	<0.0001	19F	4	0.5				

MIC values predicted from PBP type (Metcalf et al., 2016a, Metcalf et al., 2016b, Li et al., 2016) as defined at: http://www.cdc.gov/streplab/mic-tables.html.

PEN = penicillin; TAX = cefotaxime; CFT = ceftriaxone; AMO = amoxicillin; CFX = cefuroxime; MER = meropenem.

a New PBP type with MICs to PEN and TAX determined by Etest.

PBP types 15:12:18 and 13:11:16 were common resistant types within the observed lineages in the US as well (Li et al., 2016). PBP type 15:12:18 has been highly associated with the ST81 and ST156 lineages within the US since 1998, while 13:11:16 became predominant alongside 19A/ST320 during the 2000s. PBP type 45:12:63 was also observed in the US in association with 14/ST156, but only prior to PCV7 introduction (6, 8, unpublished data). PBP type 13:16:20, in association with 19F/ST1421, appeared to undergo a clonal expansion in Peru after PCV7 introduction, with the proportion of representative isolates doubling from the 2006–2009 period to the 2010–2011 period; this PBP type has been previously observed in only one isolate in the US (Li et al., 2016), also in association with 19F/ST1421.

### 3.4 Pilus genes

In *S. pneumoniae*, pili are encoded by two different pathogenicity islets, type 1 (PI-1) and type 2 (PI-2). The PI-1, and particularly the RrgA subunit, has been shown to not only contribute to adherence and virulence, but also to stimulate the host inflammatory response (Barocchi et al., 2006). The PI-2 has also been shown to contribute to adherence, but in a less effective manner than PI-1 (Bagnoli et al., 2008). Of the 133 pre-PCV7 isolates, 52% of isolates were positive for PI-1 or PI-2 type pili (inferred by detection of *rrgA* or *pitB* pilus subunit genes): 57 (42.9%) were solely PI-1+, 2 (1.5%) were PI-2+, and 11 (8.3%) were positive for both PI-1 and PI-2. While 45% of the 79 post-PCV7 isolates were positive for 1 or both determinants: 21 (26.6%) were PI-1+, 1 (1.3%) was PI-2+, and 14 (17.7%) CC1421 isolates were positive for both. The changes in prevalence of isolates carrying PI-1 or PI-1 + PI-2 were statistically significant (p = 0.005 and p = 0.003, respectively). The prevalence of PI-1 pre-PCV7 introduction among our study isolates was higher than what has been previously reported (Barocchi et al., 2006, Aguiar et al., 2008), which can be explained by its association with ST156 among these isolates. In the US, a broad array of serotypes, encompassing strains sharing 4 or more MLST alleles with ST156, are highly associated with PI-1 (6, unpublished data).

**Fig. 3:**
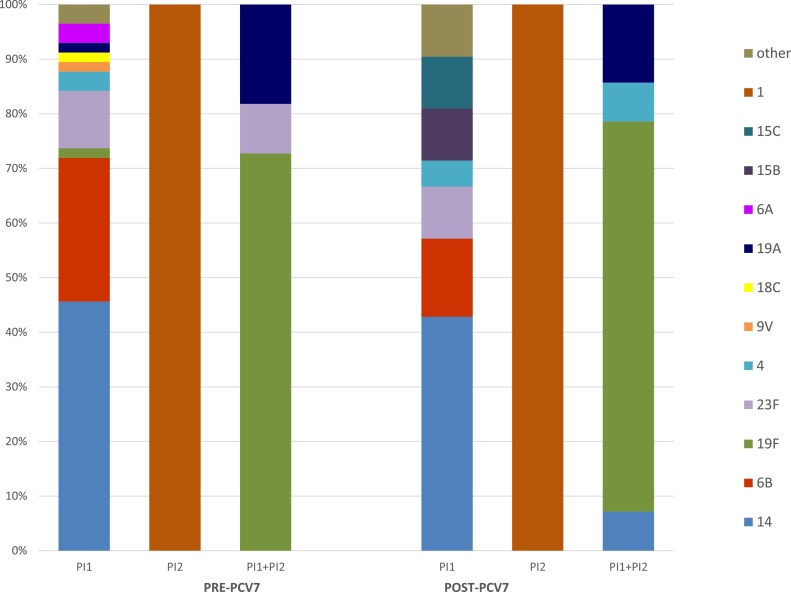
Serotype distribution of isolates positive for the presence of pilus loci, before and after PCV7 introduction.

There was a strong correlation between the presence of one or both of these pilus loci with certain clonal complexes, with the majority of isolates (52.6% pre-PCV7, 61.1% post-PCV7) belonging to either CC156 or CC1421 (Table 3). Among those isolates in CC156, almost all belonged to serotype 14 (93% pre-PCV7, 100% post-PCV7), and all of them carried PI-1 only. All CC1421 isolates were positive for both PI-1 and PI-2 and were serotype 19F or 19A (both pre and post-PCV7). All of the PI-2-positive isolates belonged to ST615 (serotype 1) (Fig. 3). These results point to a clonal expansion of the pilus genes, consistent with previous reports where PI-1 and PI-2 have been shown to be clonally distributed (Metcalf et al., 2016b, Bagnoli et al., 2008, Aguiar et al., 2008, Basset et al., 2007); with a strong association between PI-1 and STs related to PCV7 serotypes (Metcalf et al., 2016b, Aguiar et al., 2008, Basset et al., 2007). The presence of PI-1 has also been associated with resistance to antimicrobials (Aguiar et al., 2008), as a consequence of the association between PI-1 and specific ST/CCs. Among the isolates in this study, all ST156 isolates were non-susceptible to penicillin and resistant to cotrimoxazole, all ST320 isolates were non-susceptible to all 6 *β*-lactams tested and resistant to at least 3 other antimicrobial classes, and in total 12 of the 14 ST1421 isolates were non-susceptible to penicillin and resistant to at least 3 other antimicrobial classes.

**Table 3:** Association between ST/CC and the presence of a pilus locus.

	Pre PCV-7					Post PCV-7				
ST/CC	PI1	PI2	PI1 + PI2	% of ST	p-value	PI1	PI2	PI1 + PI2	% of ST	p-value
CC156	27			93.1	<0.0001	9		1	81.8	<0.0001
CC1421			10	100.0	<0.0001			12	100.0	<0.0001
CC90	7			87.5	0.006	1			100.0	**
ST242	5		1	83.3	0.03	2			66.6	**
CC1121	4			80.0	**					
ST615		2		100.0	<0.0001		1		100.0	<0.0001
CC135	3			75.0	**					
ST3669						4			100.0	0.002

*Not significant at α = 0.05.

In conclusion, these isolates show considerable genetic diversity and suggest widespread antimicrobial resistance among pneumococcal strains circulating in Lima, Peru. WGS-based serotype determination was accurate and in concordance with serological results, while antimicrobial resistance prediction results were confirmed by testing on several isolates (Etest and broth microdilution) and matched previously reported rates. Thus, the CDC’s *Streptococcus* lab pneumococcal typing pipeline performed as well on these non-US IPD isolates as it previously had on US IPD isolates.

The introduction of PCV7 in 2009 had a marked effect on ST distribution early on. The proportion of PCV7 serotypes decreased significantly from 66.9% to 50.6% post PCV7 introduction (p = 0.01), but a clear replacement by non-vaccine types was not observed, as has been reported for 19A in other countries (Hicks et al., 2007; Moore et al., 2008; WHO, 2010), nor a clonal shift within any of the vaccine serotypes. Instead, we observed the apparent expansion of a 19F/ST1421 lineage with predicted *β*-lactam resistance (PBP type 13:16:20) and carrying resistance determinants against four additional antimicrobial classes. It is important to note that this study had two major limitations: the small number of samples tested and the limited time post-PCV7 introduction covered by the study (2010–2011). It is possible that in the years since, others clones have emerged as PCV coverage increased.

The introduction of PCV10 in late 2009 is unlikely to have had an incremental effect on the prevalence and composition of strains circulating in Lima, as the three additional serotypes included in PCV10 (1, 5, 7F) were already uncommon (3.8% of post-PCV7 isolates), plus PCV10 has been shown to be less immunogenic than PCV7 against the original seven serotypes (Vesikari et al., 2009). Introducing PCV13 in Peru would potentially have a more significant effect, as this vaccine includes serotypes 3 and 19A, both of which increased after PCV7 introduction.

## Disclaimer

The findings and conclusions in this report are those of the authors and do not necessarily represent the official position of the Centers for Disease Control and Prevention.
