# Supplementary material for: Key features of invasive pneumococcal isolates recovered in Lima, Peru determined through whole genome sequencing
Source: Int J Med Microbiol. 2017 Oct;307(7):415–21. doi: 10.1016/j.ijmm.2017.07.008 (PMC5615106; doi:10.1016/j.ijmm.2017.07.008)
Supplement: Supplementary file 1 [file mmc1.docx]

Supplemental Table S1. European Nucleotide Archive Accession numbers.

| **Isolate ID** | **Accession Number** |
| --- | --- |
| GPS_P2196 | ERR433726 |
| GPS_P2197 | ERR434021 |
| GPS_P2198 | ERR1422402 |
| GPS_P2199 | ERR434022 |
| GPS_P2200 | ERR433727 |
| GPS_P2201 | ERR433661 |
| GPS_P2202 | ERR1422403 |
| GPS_P2203 | ERR433728 |
| GPS_P2204 | ERR433662 |
| GPS_P2205 | ERR433729 |
| GPS_P2206 | ERR433730 |
| GPS_P2207 | ERR433731 |
| GPS_P2208 | ERR433732 |
| GPS_P2209 | ERR434023 |
| GPS_P2210 | ERR1422404 |
| GPS_P2211 | ERR434024 |
| GPS_P2212 | ERR434025 |
| GPS_P2213 | ERR433948 |
| GPS_P2214 | ERR434026 |
| GPS_P2215 | ERR433898 |
| GPS_P2216 | ERR433733 |
| GPS_P2217 | ERR1422405 |
| GPS_P2218 | ERR434027 |
| GPS_P2219 | ERR434028 |
| GPS_P2220 | ERR433897 |
| GPS_P2221 | ERR460205 |
| GPS_P2222 | ERR433896 |
| GPS_P2223 | ERR433895 |
| GPS_P2224 | ERR433894 |
| GPS_P2225 | ERR433893 |
| GPS_P2226 | ERR425500 |
| GPS_P2227 | ERR433664 |
| GPS_P2228 | ERR1422406 |
| GPS_P2229 | ERR425501 |
| GPS_P2230 | ERR433892 |
| GPS_P2231 | ERR433891 |
| GPS_P2232 | ERR433665 |
| GPS_P2233 | ERR425502 |
| GPS_P2234 | ERR1422407 |
| GPS_P2235 | ERR433666 |
| GPS_P2236 | ERR433667 |
| GPS_P2237 | ERR433889 |
| GPS_P2238 | ERR1422408 |
| GPS_P2239 | ERR433734 |
| GPS_P2240 | ERR433887 |
| GPS_P2241 | ERR434029 |
| GPS_P2242 | ERR1422409 |
| GPS_P2243 | ERR434031 |
| GPS_P2244 | ERR425503 |
| GPS_P2245 | ERR1422411 |
| GPS_P2246 | ERR1422412 |
| GPS_P2248 | ERR1422414 |
| GPS_P2249 | ERR1422415 |
| GPS_P2250 | ERR1422416 |
| GPS_P2251 | ERR425504 |
| GPS_P2252 | ERR434033 |
| GPS_P2253 | ERR434034 |
| GPS_P2254 | ERR433670 |
| GPS_P2255 | ERR1422417 |
| GPS_P2256 | ERR433884 |
| GPS_P2257 | ERR433671 |
| GPS_P2258 | ERR433672 |
| GPS_P2259 | ERR425505 |
| GPS_P2260 | ERR433673 |
| GPS_P2261 | ERR433674 |
| GPS_P2262 | ERR433883 |
| GPS_P2263 | ERR425506 |
| GPS_P2264 | ERR433675 |
| GPS_P2265 | ERR425507 |
| GPS_P2266 | ERR433882 |
| GPS_P2267 | ERR433676 |
| GPS_P2268 | ERR1422418 |
| GPS_P2269 | ERR1422419 |
| GPS_P2270 | ERR1422420 |
| GPS_P2271 | ERR1422421 |
| GPS_P2272 | ERR433880 |
| GPS_P2273 | ERR425509 |
| GPS_P2274 | ERR1422422 |
| GPS_P2275 | ERR434036 |
| GPS_P2276 | ERR425510 |
| GPS_P2277 | ERR433679 |
| GPS_P2278 | ERR433680 |
| GPS_P2279 | ERR449189 |
| GPS_P2280 | ERR434037 |
| GPS_P2281 | ERR433879 |
| GPS_P2282 | ERR433878 |
| GPS_P2283 | ERR433877 |
| GPS_P2284 | ERR434038 |
| GPS_P2285 | ERR434039 |
| GPS_P2286 | ERR433876 |
| GPS_P2288 | ERR433949 |
| GPS_P2289 | ERR433735 |
| GPS_P2290 | ERR433681 |
| GPS_P2291 | ERR433682 |
| GPS_P2292 | ERR1422423 |
| GPS_P2293 | ERR425511 |
| GPS_P2294 | ERR433875 |
| GPS_P2295 | ERR433683 |
| GPS_P2296 | ERR1422424 |
| GPS_P2297 | ERR434043 |
| GPS_P2298 | ERR1422425 |
| GPS_P2299 | ERR433874 |
| GPS_P2300 | ERR433684 |
| GPS_P2301 | ERR1422426 |
| GPS_P2302 | ERR425514 |
| GPS_P2303 | ERR434044 |
| GPS_P2304 | ERR433873 |
| GPS_P2305 | ERR433872 |
| GPS_P2306 | ERR433871 |
| GPS_P2307 | ERR1422427 |
| GPS_P2308 | ERR433870 |
| GPS_P2309 | ERR433869 |
| GPS_P2310 | ERR425515 |
| GPS_P2311 | ERR433868 |
| GPS_P2312 | ERR1422428 |
| GPS_P2313 | ERR433867 |
| GPS_P2314 | ERR433866 |
| GPS_P3356 | ERR434046 |
| GPS_P3357 | ERR434047 |
| GPS_P3358 | ERR433865 |
| GPS_P3359 | ERR433737 |
| GPS_P3360 | ERR425516 |
| GPS_P3361 | ERR434048 |
| GPS_P3362 | ERR434049 |
| GPS_P3363 | ERR1422555 |
| GPS_P3364 | ERR433685 |
| GPS_P3365 | ERR434051 |
| GPS_P3366 | ERR460206 |
| GPS_P3367 | ERR425517 |
| GPS_P3368 | ERR434052 |
| GPS_P3369 | ERR433686 |
| GPS_P3370 | ERR434053 |
| GPS_P3371 | ERR433864 |
| GPS_P3372 | ERR425518 |
| GPS_P3373 | ERR1422556 |
| GPS_P3374 | ERR425519 |
| GPS_P3375 | ERR433950 |
| GPS_P3376 | ERR1422557 |
| GPS_P3378 | ERR433687 |
| GPS_P3379 | ERR433688 |
| GPS_P3380 | ERR425520 |
| GPS_P3381 | ERR1422558 |
| GPS_P3382 | ERR1422559 |
| GPS_P3383 | ERR433862 |
| GPS_P3384 | ERR433739 |
| GPS_P3385 | ERR433740 |
| GPS_P3386 | ERR434054 |
| GPS_P3387 | ERR433951 |
| GPS_P3388 | ERR433741 |
| GPS_P5902 | ERR1422619 |
| GPS_P5903 | ERR434056 |
| GPS_P5904 | ERR434057 |
| GPS_P5905 | ERR433742 |
| GPS_P5906 | ERR433743 |
| GPS_P5907 | ERR433952 |
| GPS_P5908 | ERR1422620 |
| GPS_P5909 | ERR433861 |
| GPS_P5910 | ERR1422621 |
| GPS_P5911 | ERR433953 |
| GPS_P5912 | ERR1422622 |
| GPS_P5913 | ERR433954 |
| GPS_P5914 | ERR433860 |
| GPS_P5915 | ERR425523 |
| GPS_P5916 | ERR1422623 |
| GPS_P5917 | ERR433744 |
| GPS_P5918 | ERR433691 |
| GPS_P5919 | ERR433745 |
| GPS_P5920 | ERR433746 |
| GPS_P5921 | ERR433692 |
| GPS_P5922 | ERR434058 |
| GPS_P5923 | ERR434059 |
| GPS_P5924 | ERR1422624 |
| GPS_P5925 | ERR434060 |
| GPS_P5926 | ERR433747 |
| GPS_P5927 | ERR433748 |
| GPS_P5928 | ERR1422625 |
| GPS_P5929 | ERR433749 |
| GPS_P5930 | ERR425525 |
| GPS_P5931 | ERR425526 |
| GPS_P5932 | ERR425527 |
| GPS_P5933 | ERR433858 |
| GPS_P5934 | ERR433693 |
| GPS_P5935 | ERR586331 |
| GPS_P5936 | ERR586332 |
| GPS_P5937 | ERR425528 |
| GPS_P5938 | ERR1422626 |
| GPS_P5939 | ERR425529 |
| GPS_P5940 | ERR433694 |
| GPS_P5941 | ERR505463 |
| GPS_P5942 | ERR505464 |
| GPS_P5943 | ERR505465 |
| GPS_P5944 | ERR505466 |
| GPS_P5945 | ERR505467 |
| GPS_P5946 | ERR505468 |
| GPS_P5947 | ERR505469 |
| GPS_P5948 | ERR505470 |
| GPS_P5949 | ERR1422627 |
| GPS_P5950 | ERR568750 |
| GPS_P5951 | ERR505472 |
| GPS_P5952 | ERR505473 |
| GPS_P5953 | ERR505474 |
| GPS_P5954 | ERR1422628 |
| GPS_P5955 | ERR1422629 |
| GPS_P5956 | ERR505477 |
| GPS_P5957 | ERR505478 |
| GPS_P5958 | ERR505479 |
| GPS_P5959 | ERR1422630 |
| GPS_P5960 | ERR505481 |
| GPS_P5961 | ERR505482 |
| GPS_P5962 | ERR505483 |
| GPS_P5963 | ERR1422631 |
| GPS_P5964 | ERR505485 |
